# 4(3*H*)-Quinazolinone: A Natural Scaffold for Drug and Agrochemical Discovery

**DOI:** 10.3390/ijms26062473

**Published:** 2025-03-10

**Authors:** Ke Chen, Shumin Wang, Shuyue Fu, Junehyun Kim, Phumbum Park, Rui Liu, Kang Lei

**Affiliations:** 1 Department of Biotechnology, The University of Suwon, Hwaseong-si 18323, Gyeonggi-do, Republic of Korea; chenke_sd@163.com (K.C.); jk819900@gmail.com (J.K.); pbpark@suwon.ac.kr (P.P.); 2 School of Pharmaceutical Sciences and Food Engineering, Liaocheng University, Liaocheng 252059, China; 13686366329@163.com (S.W.); fsyue1213@163.com (S.F.)

**Keywords:** 4(3*H*)-quinazolinone, natural products, structure–activity relationship, medicine, agrochemical

## Abstract

4(3*H*)-quinazolinone is a functional scaffold that exists widely both in natural products and synthetic organic compounds. Its drug-like derivatives have been extensively synthesized with interesting biological features including anticancer, anti-inflammatory, antiviral, antimalarial, antibacterial, antifungal, and herbicidal, etc. In this review, we highlight the medicinal and agrochemical versatility of the 4(3*H*)-quinazolinone scaffold according to the studies published in the past six years (2019–2024), and comprehensively give a summary of the target recognition, structure–activity relationship, and mechanism of its analogs. The present review is expected to provide valuable guidance for discovering novel lead compounds containing 4(3*H*)-quinazolinone moiety in both drug and agrochemical research.

## 1. Introduction

Natural products (NPs) are valuable resources produced by nature’s chemical laboratory. Historically, NPs have been important tools in treating human disease and in controlling plant diseases and insect pests [1,2,3]. In contemporary drug and agrochemical discovery, NPs continue to play an indispensable role and inspire cutting-edge research in both chemistry and biology [4,5,6,7,8,9,10,11]. Presently, two primary strategies have been extensively employed for NP-based drug and agrochemical development. The first strategy involves the development and commercialization of the NP itself [12]. For example, trabectedin [13], bialaphos [14], and spinosad [15], respectively, have been developed as antitumor agent, herbicide, and insecticide. In addition to the use of NPs directly, they also serve as lead compounds for developing novel synthetic drugs and agrochemicals (i.e., the second strategy) [16,17,18,19,20,21]. There are many publications with long lists of hundreds of NPs [22,23,24,25,26,27,28,29,30], which provide numbers of lead structures for the development of drugs and agrochemicals, and many successful instances of NPs optimization have been reported [31,32,33,34,35,36].

**Figure 1:**
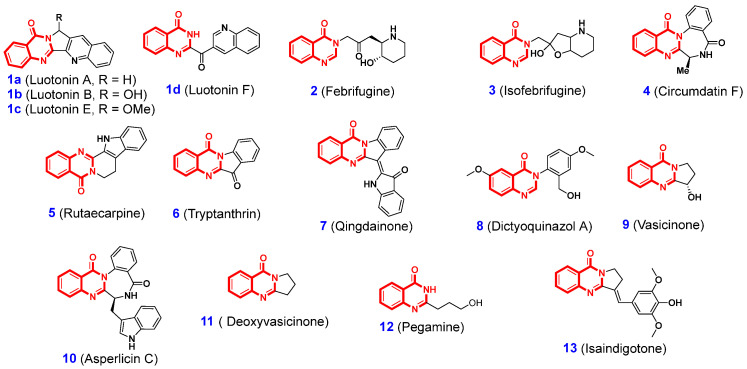
Chemical structure of some representative natural 4(3*H*)-QLOs.

**Figure 2:**
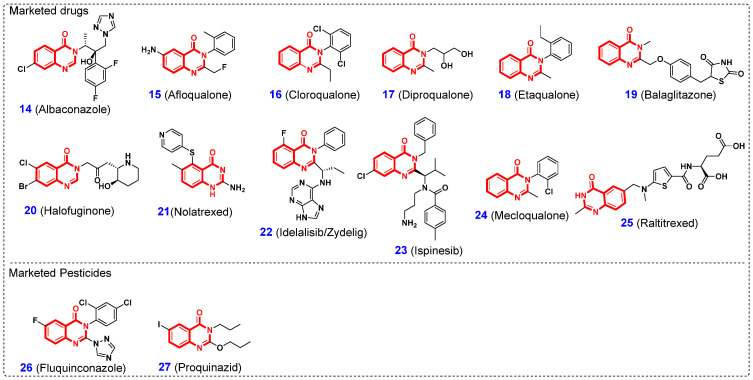
Structures of some marketed drugs and pesticides containing 4(3*H*)-QLO scaffold.

Among the known NPs, 4(3*H*)-quinazolinones (QLOs), an important class of nitrogen-containing heterocyclic compounds with a benzopyrimidone alkaloid structure, are extensively distributed in plants, microorganisms, and animals (Figure 1) [37]. Examples of such natural 4(3*H*)-QLOs are luotonins (**1**) [38,39,40], febrifugine (**2**) [41], isofebrifugine (**3**) [42], circumdatin F (**4**) [43], rutaecarpine (**5**) [44], tryptanthrin (**6**) [45,46], qingdainone (**7**) [47], dictyoquinazol A (**8**) [48], vasicinone (**9**) [49], asperlicin C (**10**) [50], deoxyvasicinone (**11**) [51], pegamine (**12**) [52], isaindigotone (**13**) [53], and so on. Extensive studies have demonstrated that natural 4(3*H*)-QLOs exhibit a broad spectrum of biological activities, including antifungal, anticancer, antiviral, radical-scavenging, antimicrobial, cytotoxic, anti-inflammatory, and antimalarial properties [22,23,26]. Consequently, the 4(3*H*)-QLO scaffold is regarded as a privileged structure for the development of novel drugs and agrochemicals. Figure 2 presents a selection of representative 4(3*H*)-QLO-containing drugs and pesticides currently available on the market for the treatment of human diseases and pest control. For example, albaconazole (antifungal agent, **14**) [54], afloqualone (GABAergic agent, **15**) [55], cloroqualone (antitussive agent, **16**) [56], diproqualone (GABAergic agent, **17**) [57], etaqualone (anxiolytic agent, **18**) [58], balaglitazone (antihyperglycemic drug, **19**) [59], halofuginone (competitive prolyl-tRNA synthetase inhibitor, **20**) [60], nolatrexed (thymidylate synthase inhibitor, **21**) [61], idelalisib (phosphoinositide 3-kinases inhibitor, **22**) [62], ispinesib (kinesin spindle protein inhibitor, **23**) [63], mecloqualone (sedative agent, **24**) [64], raltitrexed (anti-folate drug, **25**) [65], fluquinconazole (fungicide, **26**) [66], and proquinazid (fungicide, **27**) [67]. With respect to the further application of 4(3*H*)-QLOs in the discovery of new drugs and agrochemicals, it is crucial to emphasize the functional flexibility imparted by the extensive structural diversity and modifiability of compounds containing 4(3*H*)-QLOs.

This review comprehensively examines the biological activities and pharmacological significance of novel 4(3*H*)-QLOs in medical and agrochemical fields over the past six years. It includes assessments of their anticancer, anti-inflammatory, antiviral, antimalarial, antibacterial, antifungal, and herbicidal properties. Additionally, this overview profiles the structure–activity relationships (SAR) and molecular mechanisms of various promising bioactive compounds, providing valuable insights for the development of new 4(3*H*)-QLO-based drugs and agrochemicals.

## 2. Application of the 4(3*H*)-QLOs in Medicinal Chemistry

4(3*H*)-QLO is a frequently encountered scaffold in medicinal chemistry. In this part, we will highlight the most recent development of 4(3*H*)-QLOs in medicinal chemistry, including their anticancer, anti-inflammatory properties, etc.

### 2.1. Anticancer Activity

Cancer is a multifaceted global health issue that seriously threatens people’s lives. By 2030, it is projected that the annual number of new cancers diagnoses globally will reach approximately 21 million, with an estimated 17 million cancer-related deaths annually [68]. Drug therapy has become one of the important means of clinical cancer treatment. However, the complexity of designing anticancer drugs has increased over time, mainly because of growing resistance to current therapies and the poor selectivity of these drugs for tumor cells. In this context, identifying anticancer targets is considered a key link in designing highly effective, low-toxic, and highly selective cancer therapeutics. Consequently, we will concentrate on the role and efficacy of 4(3*H*)-QLOs in the development of novel anticancer drugs targeting diverse sites of action.

#### 2.1.1. Inhibition of Protein Kinases

**Figure 3:**
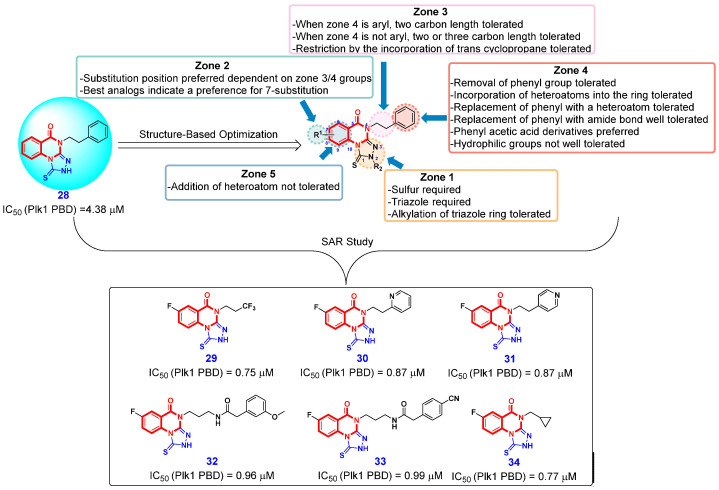
SAR of the fused 4(3*H*)-QLO scaffold for inhibition of Plk1 PBD and the structure of compounds **28**–**34**.

Polo-like kinase 1 (Plk1), an emerging anticancer therapeutic target, has been extensively investigated due to its critical role in numerous cellular functions [69]. Plk1 comprises an N-terminal kinase domain (KD) responsible for ATP-dependent catalysis, and a C-terminal polo-box domain (PBD) which is noncatalytic but functionally indispensable. Significant efforts have been devoted to developing Plk1 inhibitors that target the KD, leading to the emergence of several advanced ATP-competitive Plk1 inhibitors [70]. However, Plk1 ATP-competitive inhibitors have demonstrated limited efficacy and unacceptable dose-limiting toxicity in various preclinical and clinical trials. The PBD of Plk1 presents an alternative target for developing novel Plk1 inhibitors, potentially overcoming the challenges associated with Plk1 ATP-competitive inhibitors. Based on these facts, Lee and Jacobson et al. [71] screened a small chemical library of ~400 drug-like molecules to target Plk1 PBD. Fortunately, they identified a hit compound **28** (Figure 3), 1-thioxo-2,4-dihydro-[1,2,4]triazolo-[4,3-a]quinazolin-5(1*H*)-one, having at least threefold higher affinity with PBD than the previously characterized phosphopeptide, PLHSpT. The preliminary structure-based optimization on the 2,4-dihydro-3*H*-1,2,4-triazole-3-thione moiety of compound **28** (i.e., zone 1) revealed that the modification of thiourea moiety is not tolerated. Based on this, Lee’s team subsequently carried out a more systematic optimization campaign across various modification zones (i.e., zone 2, zone 3, zone 4, and zone 5) of compound **28**. During the optimization process, it was observed that the preferred positioning of the R_1_ group in zone 2 is contingent upon the configurations of groups in zones 3 and 4. Additionally, modifications to the benzene ring in zone 5 are not tolerated. The overall SAR results are summarized in Figure 3. The detailed SAR studies finally led to the six most potent compounds **29**–**34** having > tenfold higher inhibitory activity of Plk1 PBD than that of PLHSpT, which offer some potential lead compounds for future drug discovery against Plk1-addicted cancers.

**Figure 4:**
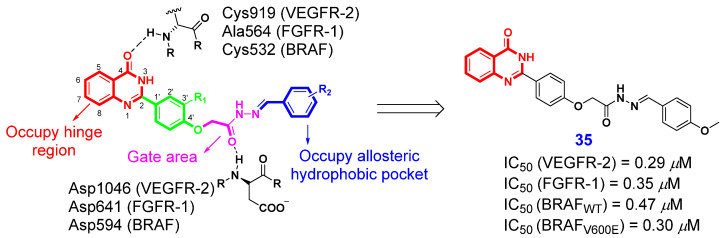
4(3*H*)-QLO-containing compound **35** as a potential antitumor agent targeting type II kinase.

Type II kinase inhibitors represent a class of non-ATP-competitors that can block the activity of the target kinase by occupying the conservative ATP binding pocket in an inactive “Asp-Phe-Gly-out” conformation [72]. The structural characteristics of type II kinase inhibitors primarily encompass: (i) a heterocyclic component that occupies the hinge region, (ii) a spacer with a bond length ranging from three to five atoms, (iii) a moiety rich in hydrogen bond donors and acceptors, and (iv) a hydrophobic component that resides within the allosteric hydrophobic back pocket [73]. Based on these structural characteristics, Abdel-Mohsen et al. [74] designed and synthesized a series of 4(3*H*)-QLOs as novel type II multi-kinase inhibitors. In these compounds, the 4(3*H*)-QLO moiety occupies the hinge region, forming stabilizing hydrogen bonds with key amino acids within the binding sites of the target kinases. Among all the synthesized derivatives, compound **35** (Figure 4) exhibited the most potent multi-kinase inhibitory activities, with IC_50_ values of 0.29 μM, 0.35 μM, 0.47 μM, and 0.30 μM for VEGFR-2, FGFR-1, BRAFWT, and BRAFV600E, respectively. Additionally, this compound exhibited excellent antiproliferative activity, achieving a mean GI_50_ of 7.16 μM across the NCI-60 cancer cell lines. It also induced G2/M phase cell cycle arrest and apoptosis in the MDA-MB-231 cell line.

**Figure 5:**
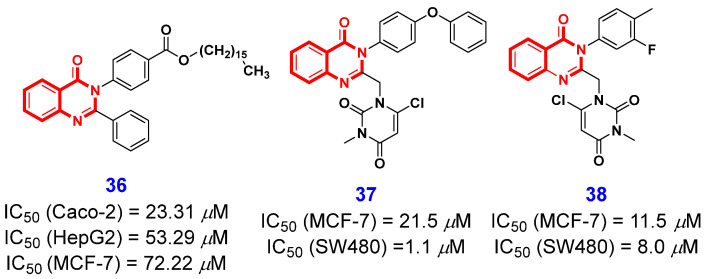
4(3*H*)-QLO-containing compounds **36**–**38** as potential antitumor agents targeting other protein kinases.

AKT is a serine–threonine kinase that plays a role in the regulation of cell growth and proliferation, as well as in cell metabolism [75]. It is reported that AKT is overexpressed in glioma, and cancers of the lung, breast, ovary, stomach, and pancreas. Therefore, the inhibition of AKT holds significant potential for cancer therapies, and substantial efforts have been devoted to developing AKT inhibitors as effective anticancer agents [76,77,78,79]. In 2020, Abdelmonsef et al. [80] synthesized a novel kind of 4(3*H*)-QLO as a potential anticancer agent targeting the AKT1 protein. In the process of conducting structure-based optimization, the authors found that the introduction of alkoxycarbonyl on the N3 benzene ring increased anticancer activity, and alkoxycarbonyl at the 4-position of N3 benzene ring is the optimal orientation. Consequently, compound **36** (Figure 5) was identified as a potential lead compound, which exhibited good inhibitory effects on Caco-2, HepG2, and MCF-7 cell lines with IC_50_ values of 23.31, 53.29, and 72.22 μM, respectively. Furthermore, the molecular docking analysis results were consistent with the findings from the in vitro study, thereby supporting the potential of compound **36** as an AKT1 inhibitor for cancer therapy.

The epidermal growth factor receptor (EGFR), as the prototypical member of the receptor tyrosine kinase family, plays a crucial role in regulating cell division and survival [81]. Overexpression of the epidermal growth factor receptor (EGFR) has been implicated in the development of various human cancers, including lungs, breast, and pancreatic cancer. Consequently, EGFR has emerged as a critical target for therapeutic interventions in these malignancies [82]. Recently, Emami and Khabnadideh et al. [83] designed and synthesized a series of substituted pyrimidine-4(3*H*)-QLOs as EGFR inhibitors and evaluated their anticancer activities against two human cancer cell lines. Notably, compounds **37** and **38** exhibited good antiproliferative effects on the SW480 cell line, with IC_50_ values of 1.1 μM and 8 μM, respectively (Figure 5). More importantly, the molecular docking revealed that compound **37** could be correctly and stably positioned at the active site of the EGFR.

#### 2.1.2. Inhibition of RecQ Helicases

The rapid proliferation of cancer cells is linked to a heightened risk of accumulating DNA damage [84,85]. RecQ helicases constitute a family of highly conserved ATP-dependent DNA helicases that play an indispensable role in preserving genomic integrity and facilitating DNA damage repair [86]. In humans, five RecQ helicases have been identified: RecQ1, bloom syndrome protein (BLM), Werner syndrome protein (WRN), RecQ4, and RecQ5 [87,88]. Among these, BLM is a critical component of DNA damage response pathways, contributing to genomic stability through mechanisms such as homologous recombination repair (HRR), telomere maintenance, and alleviation of replication stress [89,90]. Consequently, BLM has emerged as a promising target for anticancer therapies. However, research on BLM inhibitors and their biological effects remains limited [91].

**Figure 6:**
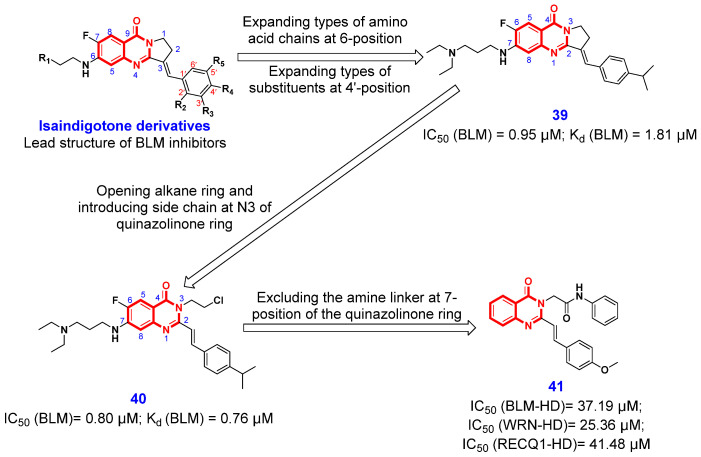
4(3*H*)-QLO-containing compounds **39**–**41** as antitumor agents targeting RecQ helicases.

**Figure 7:**
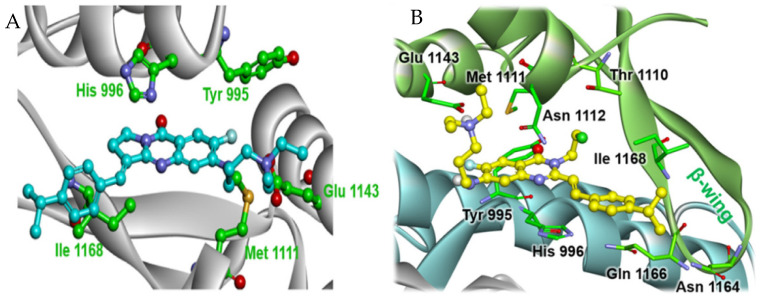
Predicted binding mode of 4(3*H*)-QLO-containing compounds **39** (**A**) and **40** (**B**) with BLM (PDB code 4CGZ).

To identify novel BLM inhibitors, Huang and Chen et al. [92] performed a screening in their in-house small molecule library and found that isaindigotone derivatives had inhibitory effects on BLM (Figure 6). Based on this, the authors screened an isaindigotone derivatives library containing 20 newly synthetic compounds and 35 reported compounds and evaluated their effects on BLM unwinding DNA. Among them, compound **39** was identified as the most potent BLM inhibitor, having a significantly inhibitory effect on BLM helicase with an IC_50_ value of 0.95 μM, which is more powerful than that of the known BLM inhibitor ML216. Furthermore, compound **39** effectively inhibited the recruitment of BLM to the DNA double-strand break site, promoted the accumulation of DNA damage repair protein RAD51, a highly conserved recombinase that serves as the primary catalyst in homologous recombination repair (HRR), and modulated the HRR process. Concurrently, this inhibitor significantly induced DNA damage response, apoptosis, and proliferation arrest in cancer cells. Subsequent isothermal titration calorimetry and molecular docking studies confirmed that compound **39** preferentially binds to the 3′-tailed duplex DNA binding pocket within the RQC domain of BLM. This binding occurs through interactions with five key amino acids, namely Tyr995, His996, Met1111, Glu1143, and Ile1168 (Figure 7A). As a continuous effort, this research group subsequently synthesized four series of new 4(3*H*)-QLO and quinoline derivatives based on the structure of compound **39**, and screened out a more potent BLM inhibitor **40** (IC_50_ = 0.8 μM) after a detailed SAR study [93]. The SAR analysis demonstrated that the 4(3*H*)-QLO scaffold is the essential structural component for BLM inhibition, whereas the amine side chain and styryl moiety exerted moderate influence. The subsequent binding studies revealed that compound **40** shares a similar binding site with compound **39** (Figure 7B) and exhibits effective binding to BLM, with a dissociation constant (*K_d_*) of 0.76 μM, which is notably stronger than compound **39** (*K_d_* = 1.8 μM). Interestingly, compound **40** potently inhibited the proliferation of the colorectal cell line HCT116 when used in conjunction with poly(ADP-ribose) polymerase (PARP) inhibitors, suggesting a synergistic lethal interaction between the BLM inhibitors and inhibitors of the DNA damage response pathway.

Given the potential for non-specific interactions between the amine linker at the 7-position of the quinazolinone ring in compound **40** (Figure 6) and the negatively charged DNA substrate, El-Hamamsy et al. [94] designed two series of novel 4(3*H*)-QLOs by eliminating the amine linker at the 7-position of the quinazolinone ring. Through detailed structural optimization, the authors found that compound **41** (Figure 6) showed strong cytotoxic activity against all examined cell lines, as well as pan-inhibitory activity against all three tested RecQ helicases (i.e., BLM, WRN, and RecQ1) with IC_50_ values of 37.1, 25.3, and 41.4 µM, respectively. The evaluation of the anticancer activity of compound **41** revealed it induced apoptosis in both HCT-116 (GI_50_ = 2.29 μM) and breast cancer cell line MDA-MB-231 (GI_50_ = 2.63 μM) cell lines, but also caused a G2/M phase cell cycle arrest in HCT-116 cells. More importantly, compound **41** was safe to the normal colon FHC cells, with a selectivity greater than 100 μM. These results suggest that compound **41** holds promise as a potential pan RecQ helicase inhibitor, demonstrating significant anticancer efficacy alongside a favorable safety profile and selectivity.

#### 2.1.3. Inhibition of Topoisomerase 1

Topoisomerase 1 (Top1) is considered an ideal target for cancer therapy owing to its critical role in modulating the degree of DNA supercoiling [95]. Several Top1 inhibitors, including camptothecin and its derivatives, have been utilized in clinical therapy [96]. However, their intrinsic chemical instability and poor solubility limit their clinical application. Consequently, there is an urgent need to develop novel Top1 inhibitors with enhanced physicochemical and pharmacokinetic (PK) properties [97].

**Figure 8:**
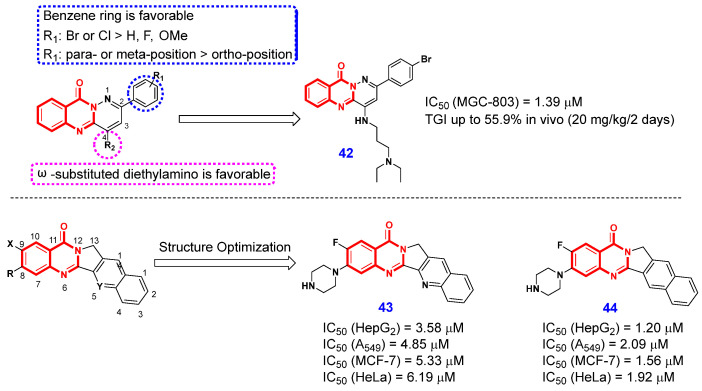
4(3*H*)-QLO-containing compound **42**–**44** as anticancer agent targeting Top1.

Given that Top1 inhibitors typically feature a poly(hetero)aromatic framework, Huang and Lyu et al. [98] designed an array of novel pyridazino[1,6-b]quinazolinone derivatives through a bicyclic fusion strategy. The detailed SAR studies demonstrated that the introduction of a benzene ring on the pyridazino[1,6-b]quinazolinone scaffold resulted in significantly enhanced anti-proliferative activity compared to alkyl or aromatic heterocyclic moieties. Furthermore, the nature and position of substituents on the benzene ring were found to substantially influence the anti-proliferative efficacy. Furthermore, introducing ω-substituted diethylamino at the 4-position of pyridazino[1,6-b]quinazolinone scaffold increased the cytotoxic activity. The systematic SAR studies culminated in the identification of compound **42** (Figure 8), which exhibited promising anticancer activity in vivo, achieving tumor growth inhibition (TGI) of up to 55.9%. In addition, agarose gel electrophoresis and molecular docking studies indicated that compound **42** exhibits potent inhibition of Top1, leading to G2 cell cycle arrest and trigger cancer cell apoptosis. These findings suggest that the pyridazino[1,6-b]quinazolinone scaffold holds promise as a novel framework for the development of new chemotherapeutic agents.

In 2021, Luo et al. [99] designed and synthesized two series of 4(3*H*)-QLOs derived from lotonin A as the lead compound and systematically evaluated their antiproliferative effects on a panel of tumor cell lines including HepG2, A549, MCF-7, and HeLa. The SAR study revealed that the introduction of piperazine at 8-position of lotonin A and the 5-position-deaza modification markedly enhanced the in vitro cytotoxicity of lotonin A against the four types of cancer. However, the introduction of a halogen at 9-position of lotonin A did not significantly affect its activity. Consequently, compounds **43** and **44** were identified as promising lead compounds, which exhibited good antiproliferative effects on HepG2, A549, MCF-7, and HeLa, with IC_50_ values of 3.58 µM, 4.85 µM, 5.33 µM, 6.19 µM, and 1.20 µM, 2.09 µM, 1.56 µM, 1.92 µM, respectively. Notably, Top1-mediated supercoiled pBR322 relaxation assay revealed that compounds **43** and **44** displayed higher Top1 inhibitory than camptothecin.

#### 2.1.4. Inhibition of Dipeptidyl Peptidase-4

Dipeptidyl peptidase-4 (DPP-4) is a glycoprotein with enzymatic activity that is expressed in various tissues and cell types. It plays a regulatory role in the expression of matrix metalloproteinases (MMPs), which are responsible for degrading the extracellular matrix, a process essential for cancer invasion and metastasis [100,101,102]. It was reported that the inhibition of DPP-4 in colorectal and lung cancers is associated with improved overall survival of the patients [103]. Consequently, DPP-4 is considered an ideal target for cancer treatment.

**Figure 9:**
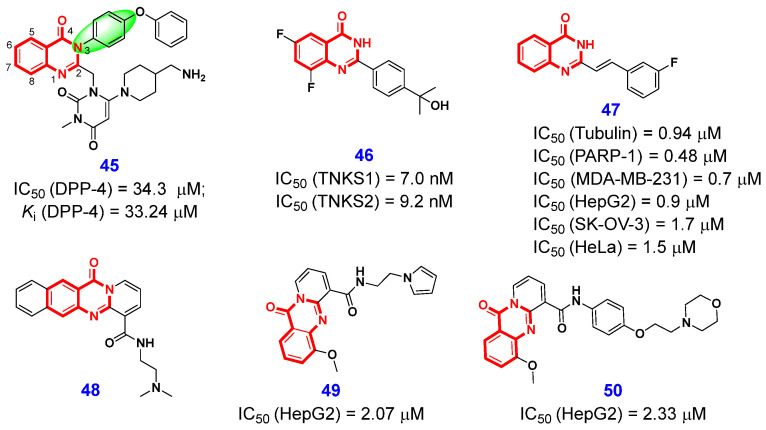
4(3*H*)-QLO-containing compounds **45**–**50** as antitumor agents.

In 2020, Khabnadideh and co-workers [104] designed a series of quinazolinone–pyrimidine derivatives by using a substructure splicing strategy for strengthening the interaction between the 4(3*H*)-QLO moiety and DPP-4 enzyme’s S_1_ pocket, as well as optimizing the benzene ring at N3-position. Among them, compound **45** (Figure 9), which acted as a competitive inhibitor, exhibited the greatest inhibition against DPP-4, with an IC_50_ value of 34.3 μM. The docking model predicted that the 4(3*H*)-QLO scaffold formed *pi–pi* interaction with Trp629 located in the S_2_’ site and interacted with Ser630 of the catalytic triad in the S_2_ pocket. The bioactivity evaluation revealed that this inhibitor exhibited significant cytotoxic activity against the HT-29 cell line, with an IC_50_ value of 10.6 µM. Additionally, it induced cell death via apoptosis or arrested cells in the G2/M phase. These results indicate that compound **45** exhibits significant DPP-4 inhibitory activity, along with potent anticancer properties, and may serve as an effective cytotoxic agent for the treatment of colorectal cancer.

#### 2.1.5. Inhibition of Tankyrase

Tankyrases (TNKS), containing two highly homologous isoforms (i.e., TNKS1 and TNKS2), are members of the PARP family [105,106]. TNKS marks axin proteins, which are concentration-limiting components of the β-catenin destruction complex, for degradation through PARsylation followed by ubiquitination mediated by the E3 ligase RNF146. It has been reported that the inhibition of tankyrase can antagonize the Wnt signaling pathway through the stabilization of axin proteins and the promotion of β-catenin degradation. Therefore, the inhibition of tankyrase activity has been considered a promising strategy for anticancer therapy in tumors dependent on the Wnt pathway [107].

In 2019, Buchstaller et al. [108] performed a high-throughput biochemical screen to identify anti-TNKS1 active compounds among nicotinamide pocket binders and found that 2-phenyl-4(3*H*)-QLO is a potential function scaffold. To improve the tankyrase potency and physicochemical and PK properties of this scaffold, a detailed structural optimization was carried out. Systematic SAR studies have demonstrated that the introduction of a 1-hydroxy-1-methyl-ethyl group at the 4′-position of the phenyl residue is a critical modification for enhancing the solubility and metabolic stability profiles of the compounds. Additionally, variations in the substitution patterns of the 4(3*H*)-QLO scaffold significantly influence both biochemical and cellular potency. Based on this information, compound **46** (Figure 9) was identified as a promising lead compound. It demonstrated enhanced activity in both biochemical and cellular assays, exhibited favorable selectivity over TNKS1 (IC_50_ = 10 nM) and TNKS2 (IC_50_ = 7 nM), and possessed acceptable pharmacokinetic properties. The crystal structure of TNKS1 in complex with compound **46** was subsequently determined, revealing that the 4(3*H*)-QLO moiety formed three hydrogen bonds with Gly1185 and Ser1221. Additionally, the terminal hydroxyl group engaged in two hydrogen-bonding interactions with water molecules. In addition, the authors detected that compound **46** was able to target the tankyrase and subsequently reduce the Wnt pathway activity in the human colorectal cancer xenograft model DLD1. These findings establish a foundation for the rational design of additional tankyrase inhibitors, aiming to develop preclinical drug-like candidates for the treatment of patients with Wnt pathway-driven tumors.

#### 2.1.6. Dual-Target Inhibitors

In recent years, the design of single-molecule multi-target antitumor drugs has attracted much attention. In comparison to single-target/single-drug or combination therapies, dual-target therapies have demonstrated the potential to mitigate side effects, decrease drug resistance, and lower treatment dosages [109,110,111,112]. In 2021, Niu and Di et al. [113] developed dual pharmacophore models of tubulin/PARP-1 based on their crystal structures and utilized these models as three-dimensional (3D) search queries to identify potential inhibitors from commercial databases. They screened out six hit compounds, which showed more than 60% inhibition to both tubulin and PARP-1 at a concentration of 10 μM. Among them, compound **47** (Figure 9) exhibited the strongest activity with ≥90% inhibition of both tubulin polymerization (IC_50_ = 0.94 μM) and PARP-1 (IC_50_ = 0.48 μM). Furthermore, the authors demonstrated that compound **47** selectively binds to tubulin by occupying a hydrophobic cavity within the colchicine binding site and forming critical hydrogen bonds with Ser904 and Gly863 in the catalytic domain of human PARP-1. Additionally, it exhibits reduced activity against other potential targets, including PI3K, PIM1, CDK4, and EGFR. The MTT assay demonstrated that compound **47** exhibited significant antiproliferative activities against a panel of human cancer cell lines, including MDA-MB-231 (human breast cancer), HepG2 (human liver cancer), SK-OV-3 (human ovarian cancer), and HeLa (human cervical cancer) cells, with respective IC_50_ values of 0.7 μM, 0.9 μM, 1.7 μM, and 1.5 μM. More importantly, compound **47** demonstrated substantial antitumor efficacy in MDA-MB-231 xenograft tumor mouse models at a dose of 5 mg/kg, while exhibiting minimal toxicity and side effects. These findings suggest its potential for development as a novel antitumor therapeutic agent.

#### 2.1.7. Inhibition of Other Anticancer Targets

A pivotal step in ribosome biogenesis is the transcription of ribosomal DNA by RNA polymerase I (Pol I). This stage of ribosome production has garnered significant attention as a promising target for the development of tumor therapeutics. Laiho et al. [114] discovered that compound **48** (Figure 9), a planar tetracyclic small molecule featuring the 4(3*H*)-quinolone core, induces proteasome-dependent degradation of RPA194 (the large catalytic subunit of the Pol I holoenzyme), thereby inhibiting Pol I transcription without causing DNA damage. Motivated by this discovery, Ranjbar et al. [115] designed and synthesized a series of novel 4(3*H*)-QLO-based small molecules to explore how structural modifications influence their efficacy across various cancer cell lines. Among them, compound **49** (Figure 9) showed a higher potency (IC_50_ = 2.07 µM) than doxorubicin against HepG2 cancer cells, while compound **50** showed comparable potency (IC_50_ = 2.33 µM) to Dox against HepG2, and the two compounds have an acceptable selection index (SI, calculated as IC_50_normalcell/IC_50_cancercell) and are safe to normal human dermal fibroblasts. Furthermore, the authors discovered that both compounds **49** and **50** are capable of activating caspase-3, which subsequently releases CAD. This process ultimately leads to the degradation of nuclear DNA and triggers apoptosis. Meanwhile, the authors observed that compounds **49** and **50** significantly inhibit the synthesis or stability of RPA194. Molecular docking studies indicated that the 4(3*H*)-QLO scaffold plays a crucial role in binding to the DNA double helix through multiple hydrogen bonds, π-stacking interactions, and van der Waals forces.

### 2.2. Anti-Inflammatory Activities

**Figure 10:**
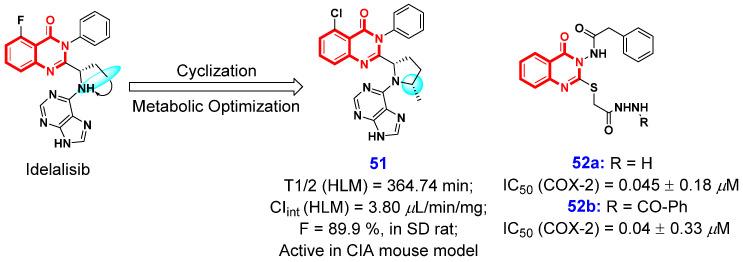
4(3*H*)-QLO-containing compounds **51** and **52** as potential anti-inflammatory agents.

Phosphoinositide 3-kinases (PI3Ks) isoforms γ and δ play crucial roles in hematopoietic cells, and have emerged as promising therapeutic targets for the treatment of inflammatory diseases [116,117,118,119,120,121]. In 2019, Bembenek et al. [122] designed a series of potent and highly selective dual inhibitors targeting both PI3Kγ and PI3Kδ. They demonstrated that incorporating a pyrrolidine linker to connect the 5,6-fused pyrrolotriazinone moiety with the purine moiety represents a viable approach for structural modification. Enlightened by their results, Chen and Yang et al. [123] identified and synthesized several potent and selective PI3Kδ inhibitors through computer-aided drug design using idelalisib as a lead compound, which is characterized by classical purine hinge binders connected to the 4(3*H*)-QLO scaffold through a pyrrolidine linker. Following comprehensive structural optimization, the authors identified that the 4(3*H*)-QLO scaffold, in conjunction with purine hinge binders, can confer selectivity and potency to PI3Kδ inhibitors. Additionally, introducing a methyl substituent on the pyrrolidine moiety can mitigate oxidative metabolism of the pyrrolidine ring while preserving or enhancing the compound’s potency. The comprehensive SAR studies led to the identification of compound **51** (Figure 10), which exhibited superior pharmacokinetic parameters in Sprague–Dawley (SD) rats, characterized by high oral bioavailability (F = 89.9%) and an appropriate half-life (T_1/2_ = 2.0 h). Notably, oral administration of compound **51** at a dose of 20 mg/kg demonstrated significant efficacy in attenuating arthritis severity in a dose-dependent manner in the collagen-induced arthritis (CIA) model, without evident toxicity. These results suggest that compound **51** holds promise as a potential therapeutic candidate for inflammatory diseases.

Cyclooxygenase (COX), a pivotal enzyme in prostaglandin biosynthesis, plays a crucial role in mediating inflammation and pain. The two isoforms of COX, namely COX-1 and COX-2, constitute the primary targets for most traditional anti-inflammatory medications [124]. In 2021, Kothayer and co-workers [125] designed and synthesized a series of anti-inflammatory quinazolinone derivatives to develop new selective COX-2 inhibitors. Among them, compounds **52a** and **52b** (Figure 10) all exhibited potent and selective COX-2 inhibitory activities, with IC_50_ values of 0.045 and 0.040 μM. Furthermore, compound **52a** demonstrated comparable in vivo anti-inflammatory activity to both ibuprofen and celecoxib, and it was more effective than indomethacin. Additionally, compound **52b** exhibited superior analgesic activity compared to celecoxib, while compound **52a** showed the highest analgesic efficacy, completely abolishing the pain response in acetic acid-induced writing in mice.

### 2.3. Antiviral Activities

**Figure 11:**
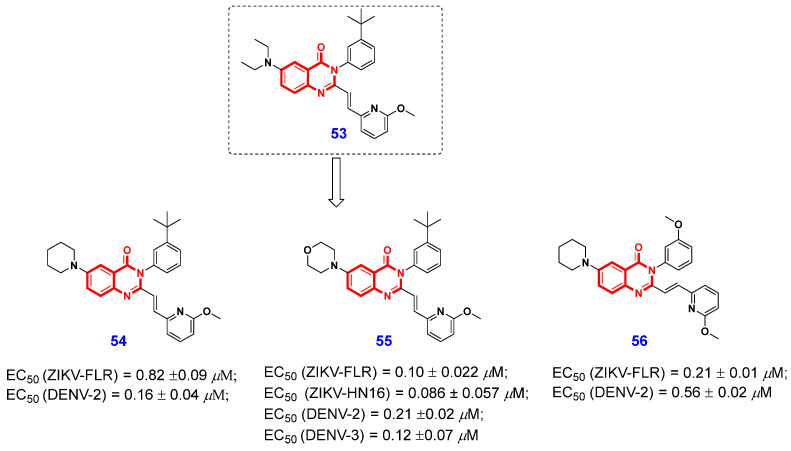
4(3*H*)-QLO-containing compounds **53**–**56** as potential antiviral agents.

Zika virus (ZIKV) and its closely related dengue virus (DENV) represent significant human pathogens, responsible for approximately 100 million symptomatic infections annually [126,127]. These viral infections can lead to severe and potentially life-threatening conditions such as microcephaly in neonates, Guillain-Barré syndrome [128], and dengue hemorrhagic fever and shock syndrome. To date, apart from mosquito control measures, no specific antiviral agents have been developed for the prevention or treatment of ZIKV and DENV infections. Therefore, there is an urgent need to find effective antiviral agents against ZIKV and DENV. In 2023, Song et al. [129] screened ~1000 compounds in their in-house chemical library and identified 2,3,6-trisubstituted 4(3*H*)-QLO compound **53** (Figure 11) as a potent anti-ZIKV agent through phenotypic screening, which can inhibit the ZIKV replication by 99.9% at 10 μM and 68% at 1 μM. The authors subsequently designed and synthesized a series of 4(3*H*)-QLO derivatives based on the structure of compound **53** and conducted a systematic study of their structure–activity relationships (SAR). The detailed SAR results revealed that an *N*-atom directly attaching to the 6-position of the 4(3*H*)-QLO scaffold provides a significant activity enhancement, and the 6-membered piperidin-1-yl or morpholin-4-yl is the most favored. The dose-dependent testing against multiple strains of ZIKV and DENV revealed that the newly synthesized compounds **54**, **55**, and **56** (Figure 11) demonstrated broad-spectrum and potent antiviral activities against both ZIKV and DENV, achieving EC_50_ values as low as 86 nM. Notably, these compounds exhibited no significant cytotoxicity across various mammalian and mosquito cell lines. The results indicate that these compounds have the potential to serve as lead compounds for the development of drugs targeting ZIKV and DENV infections.

### 2.4. Antimalarial Activity

**Figure 12:**
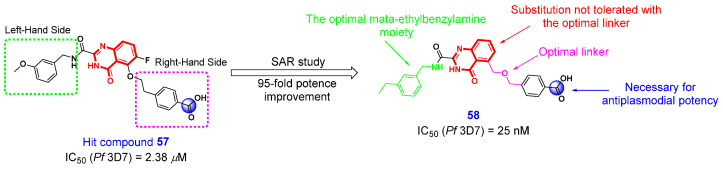
4(3*H*)-QLO-containing compounds **57** and **58** as potential antimalarial agents.

Malaria, a parasitic disease caused by *Plasmodium* parasites, is transmitted to humans via the bites of infected mosquitoes and continues to pose a significant public health challenge [130]. Five species of *Plasmodium* are known to cause this disease, with *Plasmodium falciparum* being the most lethal species. Currently, the treatment of malaria caused by *P. falciparum* predominantly depends on artemisinin-based combination therapies (ACTs). However, the widespread use of these therapies has contributed to the emergence and spread of resistance to existing antimalarial drugs. Consequently, there is an urgent imperative to identify novel antimalarial compounds to address the existing resistance. In 2021, Laleu et al. [131] performed a phenotypic high-throughput screen to search for novel antimalarials and discovered hit compound **55** (Figure 12) containing 4(3*H*)-QLO moiety that was previously reported as a selective matrix metalloproteinase-13 inhibitor [132]. With compound **57** as a lead compound, the authors subsequently performed systematic structural optimization and SAR studies. In the process of preliminary optimization, they found that the carboxylic acid group is necessary for antiplasmodial potency, and the *meta*-ethylbenzylamine moiety is the potential optimal orientation on the left-hand side of 4(3*H*)-QLO scaffold. Further, SAR studies revealed that 4-(methoxymethyl)benzoic acid moiety is the optimal orientation on the right-hand side of the 4(3*H*)-QLO scaffold. Through comprehensive structural optimization, they identified a potent compound **58** that demonstrated acceptable ligand efficiency and lipophilic efficiency. This compound exhibited 95-fold greater potency compared to the initial hit compound **57** against laboratory-resistant strains of malaria in vitro, and showed no cross-resistance with known antimalarial agents. Furthermore, the in vivo assays revealed that compound **58** possessed suboptimal DMPK and physicochemical properties, but it still showed oral efficacy in a dose-dependent manner in a murine model of human malaria. Above all, this 4(3*H*)-QLO-bearing compound **58** serves as a valuable reference for the discovery of novel antimalarial agents, potentially offering significant advantages in malaria treatment and addressing the challenge of existing drug resistance.

### 2.5. Antibacterial Activity

Pathogenic bacteria pose significant threats to human health due to their association with multiple diseases, including tuberculosis (TB) and related inflammatory conditions. Additionally, antimicrobial resistance poses an important global health challenge [133]. Therefore, the discovery of antibacterial agents is of paramount importance. Compounds bearing 4(3*H*)-QLO scaffolds, which exhibit broad-spectrum bioactivities, offer promising alternatives for addressing these diseases.

**Figure 13:**
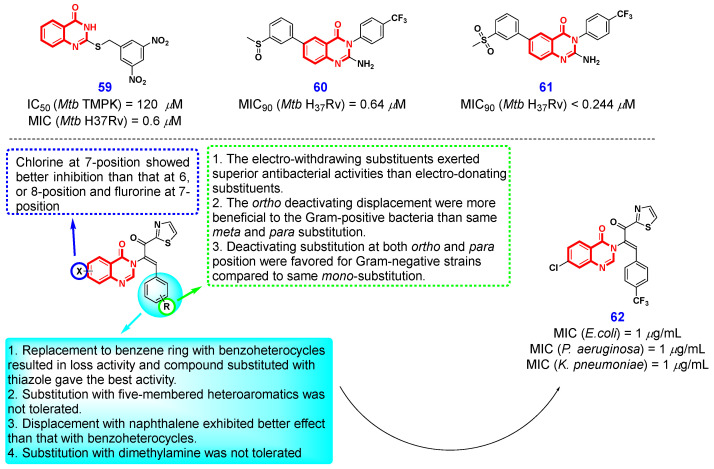
4(3*H*)-QLO-containing compounds **59**–**62** with antibacterial activity.

*Mycobacterium tuberculosis* (*Mtb*), a prevalent pathogenic bacterium, is responsible for causing tuberculosis (TB). Infection with Mtb results in approximately 10 million new TB cases globally each year [134]*. Mtb* thymidylate kinase (TMPK) is a critical enzyme essential for the survival of *Mtb*, as it plays a pivotal role in the synthesis of thymidine 5′-triphosphate, a vital component of DNA. Consequently, TMPK represents an attractive target for the development of anti-tuberculosis drugs [135,136]. In 2021, Calenbergh et al. [137] synthesized a series of 4(3*H*)-QLO analogs structurally derived from an earlier cyanopyrimidone-type *Mtb*TMPK inhibitor, as well as evaluating their *Mtb*TMPK inhibitory potency and antimycobacterial activity [138]. The preliminary in vitro bioassay results demonstrated that compound **59** (Figure 13) exhibited significant antimycobacterial activity against *Mtb* H37Rv, with a MIC value of 0.6 μM in 7H9 medium, while showing no cytotoxic effects on MRC-5 fibroblasts. Encouraged by this result, the authors further optimized the structure of **59** and systematically studied the structure–antimycobacterial activity relationship, which revealed the nitro group on the benzene ring of the (*S*)-benzyl side chain was necessary for antimycobacterial activity, and 3,5-dinitrophenyl pattern was the optimal orientation. Notably, a significant reduction in antimycobacterial activity was observed against mutants impaired in cofactor F_420_ biosynthesis, cofactor reduction, or deazaflavin-dependent nitroreductase activity. This suggests that the antimycobacterial efficacy of compound **59** is dependent on the reductive activation of the 3,5-dinitrobenzyl moiety.

In 2020, Chibale et al. [139] discovered a new class of 4(3*H*)-QLOs active against *Mtb* through phenotypic whole-cell assays and identified the 2-amino-4(3*H*)-QLO compound **58** (Figure 13) as a promising compound. The preliminary structural optimization on the left-hand side benzene ring of compound **60** demonstrated that electron-withdrawing, lipophilic substituents were highly beneficial for antimycobacterial activity, whereas electron-donating groups were not well-tolerated. Further structure-based optimization on the right-hand side of compound **60** revealed that a 2-position amino group was necessary for antimycobacterial activity, and directly attached N3 phenyl ring-bearing lipophilic groups at the *para*-position were optimal for activity. The detailed SAR studies led to the discovery of another promising compound **61**, which displayed improved aqueous solubility while maintaining potency. Interestingly, the authors found that both compounds **60** and **61** exhibited promising PK parameters, but compound **61** was inactive when evaluated for *vivo* efficacy in an acute TB infection mouse model. The discrepancy between in vitro activity and in vivo efficacy can be attributed to the significant differences in carbon metabolism observed between bacteria cultured in a standard TB medium containing glycerol and those present in TB-infected lungs.

In addition to *Mtb*, other bacterial infections also impose a significant burden on public health. Consequently, the development of broad-spectrum antimicrobial agents has emerged as one of the most prominent research areas. In 2021, Zhou et al. [140] identified 4(3*H*)-QLO-triazole as a novel structural scaffold with potential antibacterial properties, specifically designed to address the challenge of antibiotic resistance. Through detailed structural optimization, they discovered that the ethylene-bridged substituents of the target molecules occupied crucial positions in inhibiting bacterial growth. The relevant SAR findings are presented in Figure 13. The systematic structural optimization resulted in the identification of a promising compound, designated as compound **62**, which demonstrated superior antibacterial efficacy against *Klebsiella pneumoniae*, *Escherichia coli*, and *Pseudomonas aeruginosa* compared to norfloxacin, with a minimum inhibitory concentration (MIC) of 1 μg/mL. Subsequent investigations into the molecular mechanism revealed that compound **62** not only compromises both the outer and inner membranes, leading to the leakage of cytoplasmic content, but also disrupts metabolic processes by inhibiting dehydrogenase activity. At the same time, it could intercalate into DNA to exert powerful antibacterial properties. Furthermore, the authors observed that compound **62** exhibited synergistic effects against certain Gram-negative bacteria when used in conjunction with norfloxacin. Collectively, these promising results offer valuable insights into the rational design and development of novel candidates aimed at combating Gram-negative bacteria.

## 3. Application of the 4(3*H*)-QLOs in Agrochemical Chemistry

Agrochemicals can be categorized into fungicides, herbicides, insecticides, acaricides, plant growth regulators, and nematicides based on their target organisms, playing a crucial role in ensuring food security and maintaining crop quality [141]. In this section, we will detail the application of representative 4(3*H*)-QLOs in the agrochemical field, focusing on four primary categories: antifungal, antibacterial, antiviral, and herbicidal activities.

### 3.1. Antifungal Activities

Plant diseases caused by phytopathogenic fungi have resulted in significant yield losses for agricultural and horticultural crops on a global scale, posing a serious threat to global food security and public health [142]. The immense loss caused by phytopathogenic fungi has led to the wide application of various antifungal agents. However, the misuse and overuse of many traditional fungicides for a long time have caused toxicity, altered PK, and ever-rising resistance [143]. Consequently, there is an urgent need to develop novel fungicides that possess a distinctive mechanism of action, enhanced targeting efficacy, and environmental safety.

**Figure 14:**
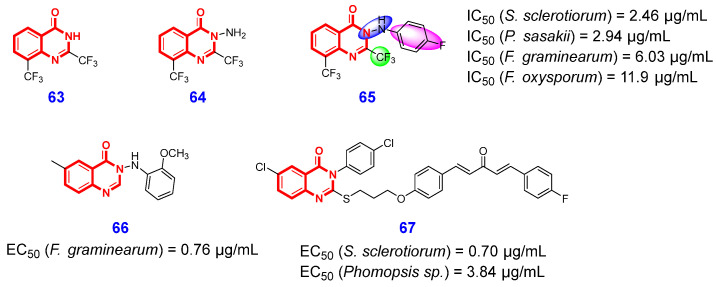
4(3*H*)-QLO-containing compounds **63**–**67** with antifungal activities.

In recent years, numerous naturally occurring 4(3*H*)-QLOs with antifungal properties have garnered significant attention from multiple research groups. In 2021, Liu et al. [144] screened the antifungal efficacy of six natural 4(3*H*)-QLOs and discovered quinazolinone and 2-methyl quinazolinone had a broader antifungal spectrum. Through preliminary structural optimization, the authors found that the introduction of an electron-withdrawing group on the 4(3*H*)-QLO scaffold was beneficial to improve antifungal activity, and compounds **63** and **64** (Figure 14) with a 2-position of the 4(3*H*)-QLO scaffold substituted by trifluoromethyl were identified as the lead compounds. Guided by this useful information, the authors continue to synthesize eight series of 4(3*H*)-QLOs and investigated the antifungal effect of N3 side-chain substituent of 4(3*H*)-QLO scaffold against phytopathogenic fungi. The SAR studies demonstrated that the hydrazine moiety and fluorophenyl substituent at the N3 side chain of the 4(3*H*)-QLO scaffold are essential for maintaining or enhancing antifungal activity. These efforts culminated in the identification of compound **65**, which demonstrated potent antifungal activity against *Sclerotinia sclerotiorum*, *Pellicularia sasakii*, *Fusarium graminearum*, and *Fusarium oxysporum*, with respective IC_50_ values of 2.46 μg/mL, 2.94 μg/mL, 6.03 μg/mL, and 11.9 μg/mL. More importantly, the *vivo* bioassay demonstrated that compound **65** exhibited comparable curative and protective efficacy (87.3% and 90.7%, respectively) against *S. sclerotiorum* relative to the positive control azoxystrobin at a concentration of 100 μg/mL. Additionally, the preliminary mechanistic analysis revealed that compound **65** can induce significant hyphal malformation and organelle damage in *S. sclerotiorum*, as well as increase cell membrane permeability.

In 2022, Xue and Yang et al. [145] designed and synthesized three series of 4(3*H*)-QLOs by modifying the rigid structure of the natural product tryptanthrin, aiming to discover novel antifungal agents that inhibit *Fusarium graminearum*. Through systematic structural optimization, compound **66** (Figure 14) was identified as a promising antifungal lead. It demonstrated significant in vitro antifungal activity against *Fusarium graminearum* (EC_50_ = 0.76 μg/mL) and *Botrytis cinerea* (EC_50_ = 1.65 μg/mL). Furthermore, the in vivo preventative efficacy of compound **66** against *F. graminearum* was measured at 59.5% at a concentration of 200 μg/mL, which is comparable to that of carbendazim. Through morphological observations, the authors detected that compound **66** induced slender and dense hyphae, distorted cell wall outlines, increased liposome numbers, and reduced mitochondria numbers. In the next year, Xue’s research group further combined 4(3*H*)-QLO scaffold with 1,4-pentadiene-3-one moiety and then synthesized a series of derivatives to screen their antifungal activities [146]. The SAR investigation revealed that the introduction of an electron-withdrawing group onto the benzene ring significantly enhances antifungal efficacy compared to an electron-donating group. Notably, compound **67** (Figure 14) demonstrated excellent broad-spectrum antifungal activity and exhibited the most potent inhibitory effects against *S. sclerotiorum* and *Phomopsis* sp., with EC_50_ values of 0.70 μg/mL and 3.84 μg/mL, respectively. More promisingly, the in vivo assay demonstrated that compound **67** exhibited superior protective and curative effects on oilseed rape, with respective efficacies of 91.7% and 87.6%, compared to azoxystrobin at a concentration of 100 μg/mL.

### 3.2. Antibacterial Activities

Plant diseases triggered by bacterial infections are the second largest cause of plant mortality and crop loss after fungal infections [147,148]. Most bacterial infections in plants are attributed to the genus *Xanthomonas*, which includes diseases such as citrus canker, rice bacterial blight, and kiwifruit canker. These can dramatically reduce crop yield and threaten food security.

**Figure 15:**
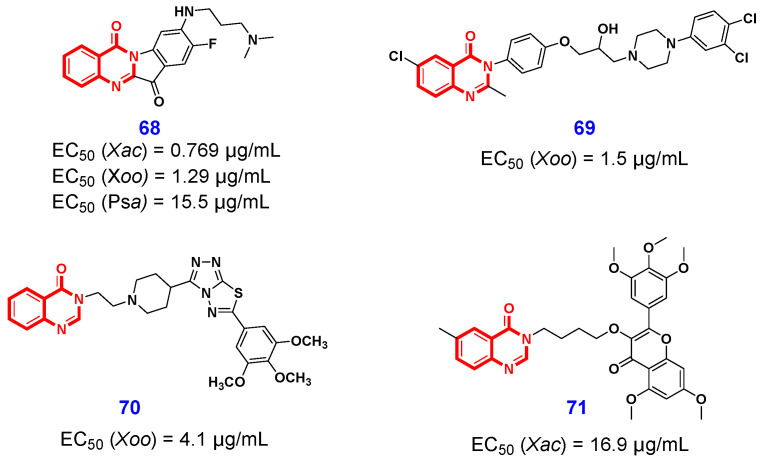
4(3*H*)-QLO-containing compounds **68**–**71** with antibacterial activities.

In 2023, Wang and Ouyang et al. [149] designed and synthesized a series of 4(3*H*)-QLOs by conjugating short-chain aliphatic amine. Delightedly, the majority of the 4(3*H*)-QLO-containing derivatives demonstrated excellent in vitro antibacterial activity. Among these, compound **68** (Figure 15) was identified as the most potent small molecule, exhibiting significantly superior bacteriostatic effects against *Xanthomonas axonopodis* pv. *citri* (*Xac*), *Xanthomonas oryzae* pv. *oryzae* (*Xoo*), and *Pseudomonas syringae* pv. *actinidiae* (*Psa*), with EC_50_ values of 0.769 μg/mL, 1.29 μg/mL, and 15.5 μg/mL, respectively. Furthermore, the vivo assays demonstrated that compound **68** exhibited significantly superior curative and protective effects compared to the commercially available pesticide thiodiazole copper (TC) at a concentration of 200 μg/mL. Preliminary mechanistic studies have demonstrated that compound **68** exhibits multiple antibacterial effects, including disruption of bacterial cell morphology, induction of reactive oxygen species (ROS) production, inhibition of bacterial growth, alteration of cell membrane permeability, and reduction in virulence. These properties contribute to improved bioavailability and enhance antibacterial efficacy. Further proteomic analysis revealed that the primary discrepancies were situated within the bacterial secretion systems pathway. As a continuing work, they further designed and synthesized two series of 4(3*H*)-QLOs by introducing isopropanolamine into the 4(3*H*)-QLO scaffold [150]. Among them, compound **67** (Figure 15) was identified as a potent antimicrobial inhibitor against *Xoo* with an EC_50_ value of 1.5 μg/mL. Through a series of comprehensive experiments, including flow cytometry, proteomics analysis, ROS assays, and defensive enzyme activity measurements, the authors observed that compound **69** may influence protein synthesis and transport within the ribosome, energy metabolism, oxidoreductase activity, and glutathione metabolism. These effects lead to an imbalance in the oxidative and reductive states of *Xoo* cells, ultimately resulting in elevated levels of ROS. In the same year, Bao et al. [151] designed and synthesized a series of novel 4(3*H*)-QLOs incorporating both the 1,2,4-triazolo[3,4-b][1,3,4]thiadiazole scaffold and a 4-piperidinyl linker. The detailed SAR studies revealed that compound **70** (Figure 15), containing a 3,4,5-tri-CH_3_Ophenyl group, can strongly inhibit *Xoo* with an in vitro EC_50_ value of 4.1 μg/mL, and effectively control rice bacterial leaf blight caused by *Xoo* with in vivo protection efficacy of 47.6% at 200 μg/mL. Furthermore, the preliminary studies on the mechanism of Anti-*Xoo* revealed that compound **70** could suppress the growth and EPS production of the pathogen *Xoo*, and alter their membrane permeability. In 2021, Xue et al. [152] disclosed a myricetin-4(3*H*)-QLO compound **71** (Figure 15) that exhibited good inhibitory activity on *Xac*, with an EC_50_ value of 16.9 μg/mL, which was better than those of the control agents bismerthiazol and TC.

### 3.3. Antiviral Activities

Plant viruses, including cucumber mosaic virus, potato virus Y, tomato chlorosis virus (ToCV), and tobacco mosaic virus (TMV), pose a significant threat to crop growth and yield, resulting in substantial economic losses globally [153,154]. The effective management of plant viral diseases remains a critical challenge in the field of plant protection. Presently, ningnanmycin (NNM) is the primary commercially available agent used for managing plant virus diseases; however, its efficacy is suboptimal [155]. Therefore, there is an urgent need to develop efficient and broad-spectrum antiviral agents.

**Figure 16:**
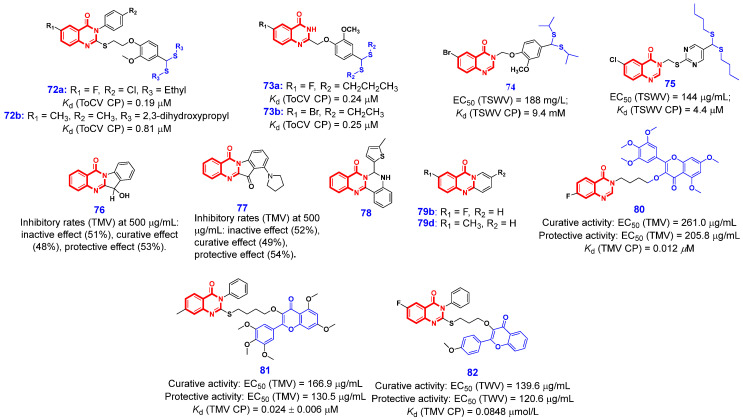
4(3*H*)-QLO-containing compounds **72**–**82** with antiviral activities.

The plant virus coat protein (CP) constitutes a critical component of viral architecture and safeguards the viral genomic nucleic acids against degradation. CP is associated with viral infection, symptom presentation, and viral migration. Additionally, it plays a role in the transcription and translation of plant viral RNAs and induces antiviral resistance [156,157]. The interaction between antiviral drugs and viral capsid proteins (CP) can effectively inhibit viral replication and assembly, thereby preventing viral infection. Consequently, CP represents a promising target for screening compounds with antiviral activity. In 2017, Song and Hu’s research group first identified dithioacetal as a potent pharmacophore with anti-plant virus activity [158]. Based on this foundation, in 2020, they designed and synthesized a series of 4(3*H*)-QLO derivatives containing a dithioacetal moiety and found that compounds **72a** and **72b** (Figure 16) exhibited strong affinities to ToCV-CP with *K*_d_ values 0.19 and 0.83 μM. Meanwhile, the two compounds **72a** and **72b** could significantly reduce the relative expression level of the ToCV-CP gene by 93.34% and 83.47%, respectively [159]. In the same year, their group further reported two novel 4(3*H*)-QLO compounds **73a** and **73b** containing dithioacetal moiety, which had strong binding capacity with the ToCV-CP with *K*_d_ values 0.24 and 0.25 μM, respectively [160]. The vivo assay demonstrated that compounds **73a** and **73b** reduced the expression levels of ToCV-CP genes by 81.05% and 87.59%, respectively. Encouraged by these results, this team continued to synthesize more dithioacetal-containing 4(3*H*)-QLO derivatives to discover new and effective antiviral agents [161]. Among them, compound **74** exhibited excellent anti-tomato spotted wilt virus (TSWV) activity in vivo with an EC_50_ value of 188 mg/L, which is superior to ribavirin, xiangcaoliusuobingmi (XCLSBM), and NNM. In addition, compound **74** showed a good binding affinity with TSWV-CP with a *K*_d_ value of 9.4 μM. Continuing this work, they further designed another series of novel 4(3*H*)-QLO pyrimidine derivatives containing dithioacetal moiety [162]. The 3D-QSAR analysis indicated that the introduction of bulky groups, hydrogen bond acceptors, and electron-withdrawing substituents at the 6-position of the QLO scaffold enhances anti-TSWV activity. In contrast, the dithioacetal moiety favors the attachment of larger and more electropositive groups. As a consequence, the optimized compound **75** exhibited excellent inactivation activity against TSWV with an EC_50_ value of 144 μg/mL and possessed better binding ability to TSWV-CP with a *K*_d_ value of 4.4 μM than those of NNM and XCLSBM.

In 2020, Wang and his coworkers discovered that tryptanthrin and its derivatives had good antiviral activities against TMV [163]. Among these compounds, compounds **76** and **77** were identified as promising antiviral lead compounds. Specifically, compound **76** demonstrated inhibitory rates of 51%, 48%, and 53% at a concentration of 500 μg/mL for inactivation, curative, and protective activities in vivo, respectively. Similarly, compound **77** exhibited inhibitory rates of 52%, 49%, and 54% under the same conditions. The preliminary mechanistic studies indicated that this compound class can inhibit virus assembly through the decomposition of the 20S capsid protein disk. In the same year, Wang’s group further designed and synthesized a series of luotonin A derivatives through ring expansion and ring contraction strategies [39]. The optimized compounds **78**, **79a**, and **79b** (Figure 16) show similar antiviral effects as NNM. Further investigation into the antiviral mechanism using transmission electron microscopy and molecular docking revealed that compound **78** can interact with tobacco mosaic virus coat protein (TMV-CP) via hydrogen bonding, inducing its polymerization and thereby inhibiting viral assembly.

In 2021, Xue’s research group discovered myricetin derivatives bearing a 4(3*H*)-QLO moiety exhibited excellent in vivo anti-TMV activity [152]. Among them, the *K*_d_ value of compound **80** (Figure 16) against TMV-CP was 0.012 μM, which was better than that of the control agent NNM. Next year, this research group further reported a myricetin-thioether-4(3*H*)-QLO fused compound **81** as the effective antiviral agent against TMV [164]. The value of *K*_d_ in the compound **81** to TMV-CP was 0.024 μM. Lately, another anti-TMV compound **82** with flavonol-4(3*H*)-QLO structure was reported by their group [165]. The EC_50_ value for the curative activity of compound **82** was determined to be 139.6 μg/mL, which is superior to that of the commercially available drug NNM. Additionally, the EC_50_ value for the protective activity of compound **82** was measured at 120.6 μg/mL, also surpassing that of NNM. Research into the antiviral mechanism indicated that compound **82** exhibits a significantly higher affinity for TMV-CP compared to NNM.

### 3.4. Herbicidal Activities

**Figure 17:**
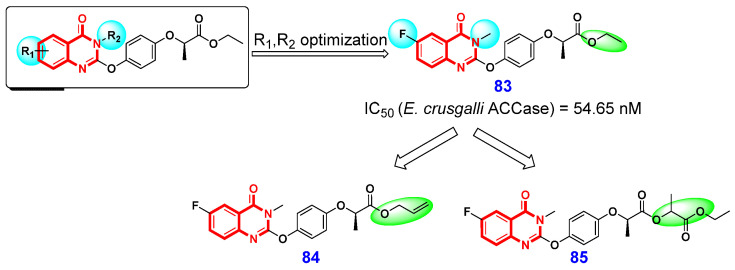
4(3*H*)-QLO-containing compounds **84**–**85** with herbicidal activities.

Although there are numerous therapeutic drugs and pesticides derived from the 4(3*H*)-QLO scaffold, the development of herbicides based on this scaffold is barely reported. In 2022, our group designed and synthesized a series of novel 4(3*H*)-QLO derivatives as herbicides [166]. The preliminary SAR studies on the 4(3*H*)-QLO scaffold revealed that the spatial position of the R group and the bulk of the R_1_ group have strongly influenced the herbicidal activity, and the (R = 6-F, R_1_ = Me) pattern was the optimal orientation. The optimized compound **83** (Figure 17) was identified as a promising herbicidal lead, which exhibited good herbicidal activities against a variety of monocotyledonous weeds with inhibition rate > 90% at 375 g ha^−1^ under pre-emergence conditions and displayed excellent crop safety to *Oryza sativa*, *Triticum aestivum*, *Gossypium* spp., and *Arachis hypogaea*. Through molecular docking and in vitro ACCase activity assay, we found that the free acid of compound **83** could bind with acetyl-CoA carboxylase (ACCase) and displayed good inhibitory activity against *E. crusgalli* ACCase with an IC_50_ value of 54.65 nM. Encouraged by these results, we further synthesized two series of 4(3*H*)-QLO derivatives based on the modification of the ester moiety of lead compound **81** [167]. The SAR studies revealed that the ester derivatives of compound **83** exhibited better herbicidal activities than those of the corresponding amide derivatives. The detailed structural optimization led to the discovery of a most potent compound **84**, which displayed broad-spectrum herbicidal activities against gramineous weeds at 187.5 g ha^−1^ under post-emergence conditions and good crop safety. Lately, we further modified the phenoxypropionate moiety through introducing lipophilic carboxylates into the ester moiety of compound **83** [168]. When its ethyl group was substituted with a branch-chain carboxylate, the resulting compound **85** was identified as a promising lead compound, which exhibited excellent herbicidal activity against gramineous weeds at 150 g ha^−1^ under post-emergence conditions and displayed higher crop safety than the commercial herbicide quizalofop-*p*-ethyl. Through a set of experiments including phenotypic observation, membrane permeability evaluation, and transcriptomic analysis, we detected that this type of compound could block the fatty acid biosynthesis pathway, damage the plasma membrane, and induce increased permeability of the cell membrane. These meaningful results made these compounds the important lead for the discovery of novel ACCase-inhibiting herbicides.

## 4. Conclusions

4(3*H*)-QLO is a scaffold of significant interest in the pharmacophore, featuring prominently in various drugs, clinical candidates, and bioactive molecules. Due to the 4(3*H*)-QLO scaffold’s diverse and crucial pharmaceutical potential, 4(3*H*)-QLO-based derivatives have been broadly developed as key lead compounds or candidates for addressing various human diseases such as cancer, inflammation, and viral and bacterial epidemics. They are also utilized in agrochemicals as pesticide molecules, such as herbicides, fungicides, and insecticides. In this review, we collected state-of-the-art investigations of the 4(*3H*)-QLO scaffold, which seeks to incorporate this scaffold into the discovery of novel drugs or green pesticides.

## Abbreviations

NPs	Natural products
QLOs	4(3*H*)-quinazolinones
SAR	Structure–activity relationship
Plk1	Polo-like kinase 1
KD	Kinase domain
PBD	Polo-box domain
AKT	Serine–threonine kinase
EGFR	Epidermal growth factor receptor
BLM	Bloom syndrome protein
WRN	Werner syndrome protein
HRR	Homologous recombination repair
*K*_d_	Dissociation constants
PARP	Poly (ADP-ribose) polymerase
Top I	Topoisomerase I
PK	Pharmacokinetic
TGI	Tumor growth inhibition
DPP-4	Dipeptidyl peptidase-4
TNKS	Tankyrases
3D	Three-dimensional
Pol I	Polymerase I
PI3Ks	Phosphoinositol 3-kinases
SD	Sprague−Dawley
T_1/2_	Half-life
CIA	Collagen-induced arthritis
COX	Cyclooxygenase
ZIKV	Zika virus
DENV	Dengue virus
TB	Tuberculosis
*Mtb*	*Mycobacterium tuberculosis*
TMPK	Thymidylate kinase
*Xac*	*Xanthomonas axonopodis* pv. *Citri*
*Xoo*	*Xanthomonas oryzae* pv*. Oryzae*
*Psa*	*Pseudomonas syringae* pv. *Actinidiae*
TC	thiodiazole copper
ToCV	Tomato chlorosis virus
TMV	Tobacco mosaic virus
NNM	ningnanmycin
CP	Coat protein
TSWV	Tomato spotted wilt virus
XCLSBM	Xiangcaoliusuobingmi
ACCase	Acetyl-CoA carboxylase
